# The value of the wireless stethoscope in patients with COVID-19 infection in a makeshift hospital

**DOI:** 10.1186/s12938-023-01136-5

**Published:** 2023-07-14

**Authors:** Ying Zhuge, Liu Rong, Lei Ye, Jiaqi Liu, Lingyun Su, Zhiping Zhang, Junshan Wang, Zhi Zhang

**Affiliations:** 1 Department of Cardiology, Shanghai General Hospital, Shanghai Jiaotong University School of Medicine (Originally Named “Shanghai First People’s Hospital”), 100 Haining Road, Shanghai, 200080 People’s Republic of China; 2 Department of Critical Care Medicine, Shanghai General Hospital, Shanghai Jiaotong University School of Medicine (Originally Named “Shanghai First People’s Hospital”), Shanghai, China; 3 Department of Nursing, Shanghai General Hospital, Shanghai Jiaotong University School of Medicine (Originally Named “Shanghai First People’s Hospital”), Shanghai, China; 4 Department of Gastroenterology, Shanghai Tenth People’s Hospital, Tongji University, Shanghai, China

**Keywords:** Wireless stethoscopes, SARS-CoV-2, COVID-19, Makeshift hospital, Auscultation

## Abstract

**Objective:**

When COVID-19 sweeps the world, traditional stethoscopes are seen as infectious agents and then the use of stethoscopes is limited especially when health providers were in their personal protective equipment. These reasons led to the ignoring of the values of stethoscopes during pandemics. This study aims to explore the value of wireless stethoscopes in patients of a makeshift hospital.

**Material and methods:**

200 consecutive hospitalized patients with confirmed SARS-CoV-2 at Lingang Makeshift Hospital in Shanghai, China, were enrolled from April 10 to May 10, 2022 (Trial Registration Number: ChiCTR2000038272,2020/9/15). They were randomly divided into two groups. In group A (*n* = 100), patients were examined without a stethoscope. In group B (*n* = 100), lung breath sounds and heart sounds were examined with a wireless stethoscope, and positive signs were recorded. The duration of cough and tachycardia symptoms, as well as emergency cases, were compared between the two groups. In addition, the pressure, anxiety, and depression of patients in the two groups were investigated using the DAS-21 questionnaire scale, to observe the psychological impact of the stethoscope-based doctor–patient communication on patients in the makeshift hospital.

**Results:**

There was no significant difference in baseline characteristics between the two groups. In group B, some significant positive signs were detected by wireless stethoscopes, including pulmonary rales and tachycardia, etc. Moreover, the therapeutic measures based on these positive signs effectively alleviated the symptoms of cough and tachycardia, which showed that the duration of symptoms was significantly shorter than that of group A (cough: 2.8 ± 0.9 vs. 3.6 ± 0.9; palpitation: 1.4 ± 0.7 vs. 2.6 ± 0.7). In particular, the number of emergency cases in group B is less than that in group A (1% vs. 3%), and the severity is lower. Notably, stethoscope-based doctor–patient communication was found to be effective in alleviating psychological measures of group B patients.

**Conclusion:**

Wireless stethoscopes in makeshift hospitals can avoid cross-infections and detect more valuable positive signs, which can help health providers make accurate decisions and relieve patients' symptoms more quickly. Moreover, stethoscope-based doctor–patient communication can diminish the psychological impacts of the epidemic on isolated patients in makeshift hospitals.

*Trial registration* This study was registered in the Chinese Clinical Trial (ChiCTR2000038272) at http://www.chictr.org.cn. http://www.chinadrugtrials.org.cn/clinicaltrials.searchlistdetail.dhtml.

## Background

Severe acute respiratory syndrome coronavirus 2 (SARS-CoV-2) has swept across the globe, creating many uncertainties in healthcare systems and societies. However, the new SARS-CoV-2 virus Omicron has undergone more changes in its prevalence, biology, and clinical characteristics, which made a lot of difficulties in dynamic “zero infection” in China before December 2022 [1, 2]. Although there is no evidence that the Omicron variant causes more severe disease or long-term infection, its high infectivity, risk of reinfection, and increased hospitalization rates require a response [3].

Building makeshift Hospitals is undoubted once an effective method to prevent the rapid spread of the virus. However, its main function is isolating the infected people with mild symptoms in large-scale construction, so the makeshift hospitals often lack many basic medical equipments, such as CT or Echo. In particular, medical staff wearing protective clothing cannot use traditional stethoscopes. As a result, in makeshift hospitals, doctors usually rely on empirical diagnosis to treat those mild cases. However, it causes potential medical risks and worsens the patients’ experience.

Fortunately, we still have an antique weapon named the “stethoscope” to arm us which is often ignored in modern medical practice. The stethoscope enables doctors to listen, touch, and diagnose patients, thus realizing the basic pillar of clinical medicine [4]. For instance, an S3 heart sound highly predicts left ventricular dysfunction and has a profound impact on the prognosis of patients with heart failure. [5]

Recently, the emergence of digital stethoscopes has advantages over traditional acoustic stethoscopes, such as ambient noise reduction, heart sound amplification, and Bluetooth transmission [6, 7]. The ability to record and transmit lung sounds and heart sounds is very useful for patients in isolation precautions. In this case, multiple health providers can listen to the heart–lung auscultation when only one person needs to enter the patient’s room [8]. And our previous attempts to auscultate the lung of patients with SARS-CoV-2 pneumonia in Wuhan showed a successful example to use a wireless stethoscope in isolated wards [9]. Kinds of crackles including fine crackles and wheezing were heard and recorded in those patients.

To evaluate the value of wireless stethoscopes in makeshift hospitals, we investigated 200 patients with mild infection in the Lingang No.2 Makeshift hospital in Shanghai. A newly designed electronic wireless stethoscope called Stemoscope was used to collect the cardiac and pulmonary auscultation results and the medical decision was then made correspondingly.

## Results

### Clinical characteristics

All 200 patients were asymptomatic or mildly infected with SARS-CoV-2, which was confirmed by Shanghai CDC, and were transferred from communities in Shanghai. Among the 200 patients, there were 100 patients in group A (41 males and 59 females) and 100 patients in group B (39 males and 61 females). The medium age was 41 years (18–79 years). Fifty-five patients were asymptomatic (23% in group A and 22% in group B), and 145 patients were mild (77% in group A and 78% in group B). The most common symptom was sore throat (39%), followed by cough (24.5%). Of the enrolled patients, 27 had comorbidities (15% in group A and 12% in group B) (Table 1).

**Table 1:** Baseline characteristics of patients infected with SARS-CoV-2

	group A	group B	*p*-value
Age(years)	46 ± 17	47 ± 17	0.61
< 65	87	85	1
≥ 65	13	15	1
Sex			
Female	59	61	1
Male	41	39	1
Comorbidities			
Diabetes	2	1	0.246
Hypertension	5	6	0.537
Cardiovascular disease	3	2	0.367
Cerebrovascular disease	2	3	0.367
Asthma	2	2	1
Mental illness	2	1	0.246
Malignancy	1	1	1
Signs and symptoms			
Asymptomatic	23	22	0.736
Cough	23	26	0.327
Runny nose	10	9	0.632
Headache	3	2	0.367
Fatigue (either mild or severe)	5	7	0.235
Sneezing	15	12	0.216
Sore throat	36	42	0.094
Fever (> 37.3℃)	5	6	0.537
Expectoration	19	21	0.482
Insomnia	3	4	0.444
Palpitation	9	10	0.632
Systolic blood pressure	123.30 ± 15.44	124.73 ± 16.74	0.985
Diastolic blood pressure	66.06 ± 11.18	68.82 ± 13.08	0.054
Pre-hospital treatment			
No treatment	21	19	0.482
Traditional Chinese medicine	56	59	0.402
NSAIDs	18	19	0.717
Hypertension drug	4	5	0.497
Diabetes drug	2	1	0.246
Antiasthmatic drugs	2	1	0.246
Hypnotic sedative drugs	3	4	0.444

### Positive signs obtained by Stemoscope

Different from group A, in group B patients, some positive signs were detected by wireless stethoscopes and were consistent with patients’ symptoms and past medical history (Table 2). Of the seven patients who were found to have dry rales, five had significant cough symptoms that were not easily relieved with cough suppressants. However, when orthoxine is administered orally, symptoms are quickly relieved. The other two patients had a history of asthma and were relieved by salbutamol. Among the three patients with moist rales, two patients were given ambroxol and one was given furosemide for acute heart failure, and all were transferred to a designated COVID-19 hospital for further treatment. Surprisingly, in group B, arrhythmias were detected by wireless stethoscope in 19% of patients, including tachycardia, bradycardia, and premature beats. In particular, one patient found to have a heart murmur was advised to have an echocardiogram. She was eventually diagnosed with an atrial septal defect after leaving the makeshift hospital.

**Table 2:** Positive signs obtained by Stemoscope and the following treatments

Positive signs	Cases	Follow-up procedure	Outcome
Tachycardia (HR > 110 bpm)	14	Metoprolol	palpitation was quickly relieved
Bradycardia (HR < 50 bpm)	1	Transfer to a designated hospital for ECG	No serious consequences
Dry rales	7	Orthoxine or Salbutamol	Cough quickly relieved
Moist rales	3	Furosemide or Ambroxol, prompt referral for further treatment	No serious consequences
Premature beat	4	Two patients were given explanatory comfort, and two were given metoprolol	Palpitation is relieved and anxiety is eliminated
Cardiac murmur	1	Echocardiography is recommended	An atrial septal defect was diagnosed

### Symptom relief efficiency

Because patients in group A adopted empiric diagnosis and treatment methods (including consultation, palpation, blood pressure measurement), they obtained fewer meaningful positive signs compared with group B which used a wireless stethoscope. Since cough and palpitation were the most common symptoms in patients in makeshift hospitals, we compared their relief time of them between group A and group B (Table 3). The results showed that patients in group B who used the wireless stethoscope experienced quicker relief of cough (2.8 ± 0.9 vs. 3.6 ± 0.9, *p* < 0.01) and palpitation (1.4 ± 0.7 vs. 2.6 ± 0.7, *p* < 0.01) symptoms.

**Table 3:** The relief time of cough and palpitation between group A and group B

	Duration time(days)		
	group A	group B	*P*-value
cough	3.6 ± 0.9	2.8 ± 0.9	0.006
palpitation	2.6 ± 0.7	1.4 ± 0.7	0.003

### Emergency events and outcomes

There were three severe events in group A, including an unexplained sudden death in one case, acute left heart failure in one case, and refractory asthma in one case. However, there were no serious acute events in group B. There was no significant difference between the two groups (*P* = 0.621). (Table 4) Except for one death, the other three patients survived when given appropriate treatment.

**Table 4:** Emergency events between two groups

Emergency events	group A	group B	
Sudden death	1	0	
Acute heart failure	1	0	
Asthma attack	1	0	
Vasovagal syncope	0	1	
total	3%(3/100)	1%(1/100)	*P* = 0.621

### Psychological assessment

**Fig. 1:**
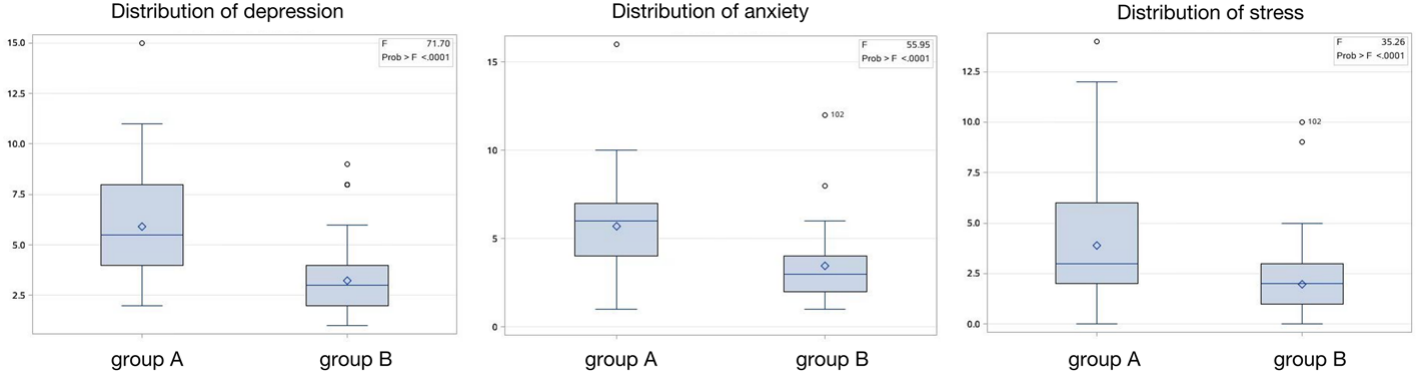
Comparison of the psychological states of patients between group A and group B. The psychological scores of depression, anxiety, and stress between group A and group B are 5.9 ± 2.6 vs. 3.2 ± 1.8 (*p* < 0.01), 3.9 ± 2.8 vs. 2.0 ± 1.6 (*p* < 0.01), and 5.7 ± 2.5 vs. 3.5 ± 1.7 (*p* < 0.01), respectively

DASS-21 questionnaire was used to investigate the psychological states of patients between group A and group B, including depression, anxiety, and stress. The data showed that the three psychological scores of group A were significantly higher than group B. In addition, the number of patients with mild depression and anxiety in group A was also more than that in group B. (Fig. 1).

## Discussion

In this study, patients with SARS-CoV-2 were successfully auscultated by using a wireless stethoscope in the makeshift hospital. Doctors who were dressed in their personal protective equipment auscultated 100 patients. And for the first time, we test the efficacy of auscultation with a wireless stethoscope in a makeshift hospital where auscultation used to be unavailable. And we found auscultation could detect some positive signs and shorten the relief time of symptoms, which could also improve the psychological states of patients.

The stethoscope has long been at the center of patient care, as well as a symbol of the physician–patient relationship. [8] However, the COVID-19 pandemic has led many people to view stethoscopes as potentially infectious agents [10, 11]. On the other hand, personal protective equipment (N95 masks, gowns, gloves, and eye protectors) for healthcare workers in contact with COVID-19 patients is also a barrier to using traditional stethoscopes. For these reasons, the use of stethoscopes in makeshift hospitals has been severely restricted. But with the development of electronic stethoscope technology, the wireless stethoscope could become a very valuable tool.

In this study, a comparative evaluation of wireless stethoscopes was performed in two hundred asymptomatic or mild patients with SARS-CoV-2 infection. Among them, 100 patients received a stethoscope examination of the hearts and lungs. Surprisingly, 30% (30/100) of these patients, who would have been considered asymptomatic or mild, were found to have positive signs by stethoscope. Moreover, after these patients received medical interventions or advice, they had relatively good outcomes.

In our study, patients with cough or palpitation symptoms were chosen with priority, because these two symptoms were more likely associated with positive signs. The results showed that the wireless stethoscope could lead to timely and accurate medical intervention of cough and palpitation, which significantly shortened the duration of symptoms. Among the patients in group B who presented with cough, 4 out of 26 (15.4%) were found to have dry rales, which might have a mechanism of variant asthma (CVA) [12]. Symptoms were quickly relieved when orthoxine or salbutamol was applied. This is consistent with previous reports of patients with CVA co-infected with SARS-CoV-2, who ultimately need to rely on hormones or salbutamol for remission [13]. In contrast, 23% (23/100) of group A patients also complained of cough, with significantly longer duration than group B (3.6 + 0.92 vs. 2.8 + 0.91 days, *P* < 0.01). This is because different pathological mechanisms of cough cannot be accurately distinguished only by the presentation of cough symptoms, which could not lead to accurate treatment [14].

In addition, for patients complaining of palpitation, Stemoscopes can identify tachycardia and premature beats easily. More importantly, the information obtained by auscultation helps in the use of drugs. However, in group A which lacked the help of Stemoscopes, doctors often choose to wait and see because they could not obtain the details of the patient's auscultatory information. And they also worried about the side effects of drugs, which led to a longer duration of symptoms than group B (2.6 + 0.68 vs 1.4 + 0.66 days, *P* < 0.01).

Although patients admitted to makeshift hospitals have been initially screened, due to a large number of patients and the relative shortage of medical resources, unexpected emergencies can occur. There were several acute cases in both groups. Although there is no significant difference, both quantity and severity seemed higher in group A than in group B (3% vs 1%, *p* = 0.621). For some high-risk patients, early medical intervention will significantly reduce the risk of exacerbation and death, including non-COVID-19 diseases [15]. From this point of view, a wireless stethoscope is a very important and indispensable tool in makeshift hospitals.

It is worth mentioning that patients with confirmed SARS-CoV-2 infection can develop several psychological consequences [16]. A survey conducted in the early days of the COVID-19 outbreak in China showed that more than half of respondents were considered to have moderate to severe psychological impact, and about one-third had moderate to severe anxiety [17]. In the makeshift hospital, patients are placed in isolation and separated from their loved ones—thus, the ability of clinicians to establish physical and emotional relationships with them is more important than ever. A stethoscope is undoubtedly an important tool for maintaining a good doctor–patient relationship. In our study, the results of the DASS-21 questionnaire showed that the scores of stress, depression, and anxiety in group B patients were significantly lower than those in group A (Fig. 1). This may be because patients feel safer when using a wireless stethoscope for physical examination. Researchers who use wireless stethoscopes can actually experience a more trusting and appreciative doctor–patient relationship.

As a non-invasive bedside tool that can quickly collect important information, the stethoscope is a practical and portable piece. In the current situation of SARS-CoV-2 ravaging the world, the advantages and disadvantages of traditional stethoscopes have been controversial. Homemade stethoscopes have even been reported for COVID-19 patients [18]. In pandemic situations, wireless stethoscope not only helps health providers auscultate but also reduces their direct contact with infected patients. This study supports the view that patients in the epidemic need not only professional medications but also physical contact between doctors and patients, and the stethoscopes integrate them.

Our study has some limitations. Firstly, when this study was launched, China had classified COVID-19 as a Category B infectious disease but treated it as a Category A disease. However, at the time of the publication of this article, China had treated it as a Category B disease. So, this scenario may no longer apply to the treatment of COVID-19, but it still works for other pandemics that might occur in the future. Secondly, because there were not too many SARS-CoV-2 patients left in Shanghai makeshift hospitals when the study started, only 200 eligible patients with confirmed SARS-CoV-2 were included in this study. Finally, although Stemoscope is designed to be waterproof and easy to sanitate, we have not studied this advantage in this clinical trial.

Although the Stemoscope used in this study was not assisted with artificial intelligence (AI), the development of machine learning (ML) and AI technology has allowed the analysis of complex data more reliable in the future, such as heart and lung sounds, abnormal rhythms, and murmurs. [19, 20]

## Conclusion

Wireless stethoscopes in makeshift hospitals can avoid cross-infections and detect more valuable positive signs, which can help health providers make accurate decisions and relieve patients’ symptoms more quickly. Moreover, stethoscope-based doctor–patient communication can diminish the psychological impacts of the epidemic on isolated patients in makeshift hospitals. In the context of a pandemic, the stethoscope is more than an important clinical tool, which also ties doctors and patients together.

## Methods

### Study design and participants

**Fig. 2:**
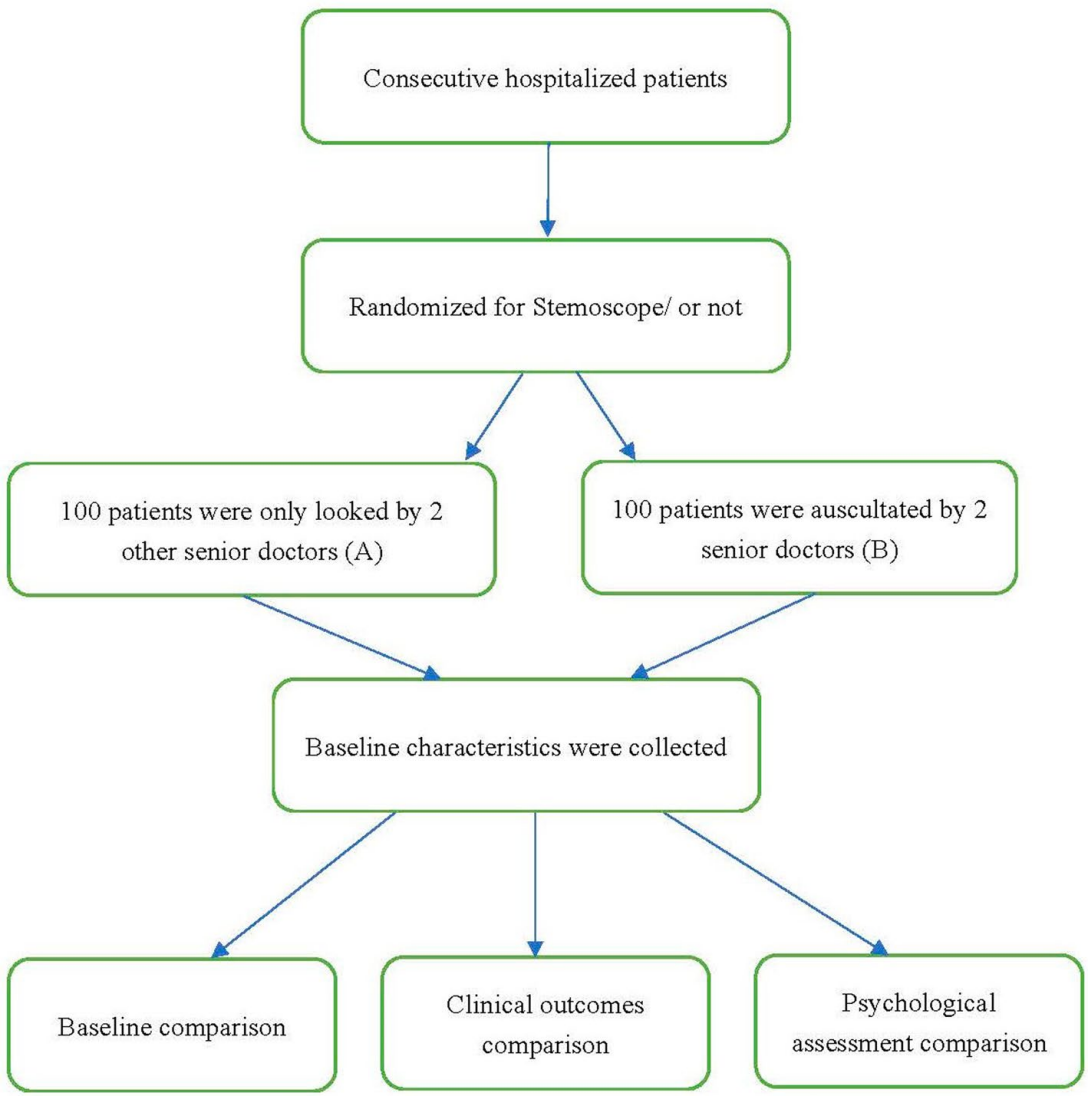
The flowchart of the study

This single-center study was approved by the institutional ethics Committee of Shanghai General Hospital affiliated with Shanghai Jiao Tong University (Approval Number 202029). All patients tested positive for SARS-CoV-2 nucleic acid and were admitted to the Lingang No.2 Makeshift Hospital. This study aimed to explore the value of auscultation in the following two groups: group A, with auscultation, and group B, without auscultation. Figure 2 shows the flowchart of the study. Patients were enrolled from April 10 to May 10, 2022, and randomly assigned to two groups: group A (*n* = 100) and group B (*n* = 100). All patients were diagnosed as mild SARS-CoV-2 infected according to the Chinese COVID-19 Diagnosis and Treatment Protocol (CDTPNCP Trial Version 9). An electronic wireless stethoscope (Stemoscope, Hulu Devices) was used to auscultate group B patients through an app installed on a smartphone and record the results, while group A patients received only empirical diagnosis and treatment.

Written or oral informed consent was obtained from the patient. The exclusion criterion was as follows: the investigator believed the patient should not be included (i.e., heart sounds or lung sounds were extremely hard to hear or hard to recognize).

### Data collection

The medical records of all SARS-CoV-2 confirmed patients were reviewed and analyzed by the subject group of Shanghai Lingang No. 2 makeshift hospital, which was taken over by Shanghai General Hospital. Clinical electronic medical records, laboratory test results, treatment, and outcome data are obtained through data collection forms. Data on gender, age, onset date, symptoms, chronic history, vital signs at admission (heart rate, respiratory rate, blood pressure), and treatment measures were also collected.

**Fig. 3:**
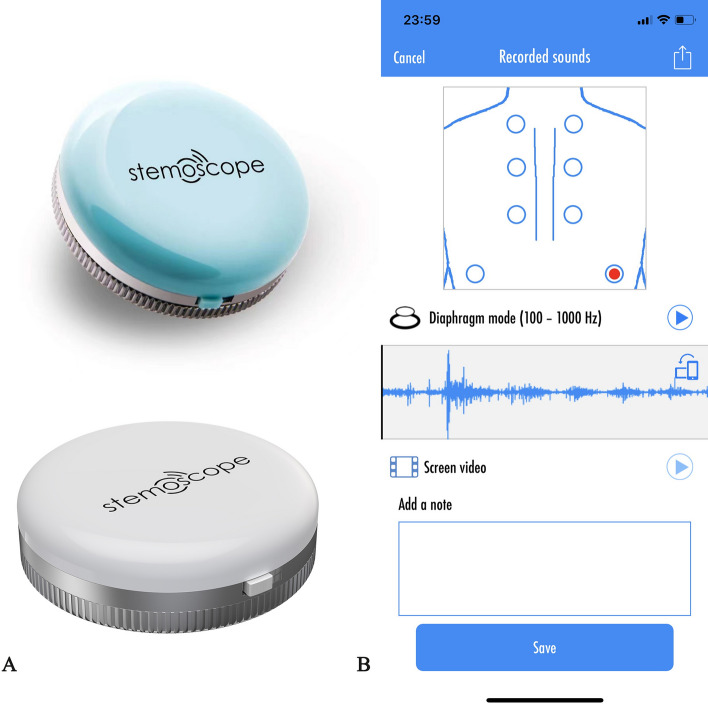
Respiratory sounds were recorded in 6 pulmonary fields bilaterally (**A**, **B**). The total record sites were 12, 2 at the basal field anterior (**A**) and posterior (**B**), 2 at the middle field anterior (**A**) and posterior (**B**), and 2 at the upper field anterior (**A**) and posterior (**B**). Heart sounds were recorded in the following 5 areas (**C**): aortic area; Erb’s point; pulmonic area; tricuspid area; mitral area

Respiratory sounds were recorded in 6 pulmonary fields bilaterally (2 at the basal field anterior and posterior, 2 at the middle field anterior and posterior, 2 at the upper field anterior and posterior) with a Stemoscope (Fig. 3A, B). Cardiac sounds were recorded in five auscultation areas (aortic area, Erb's point, pulmonic area, tricuspid area, and mitral area) (Fig. 3C).

**Fig. 4:**
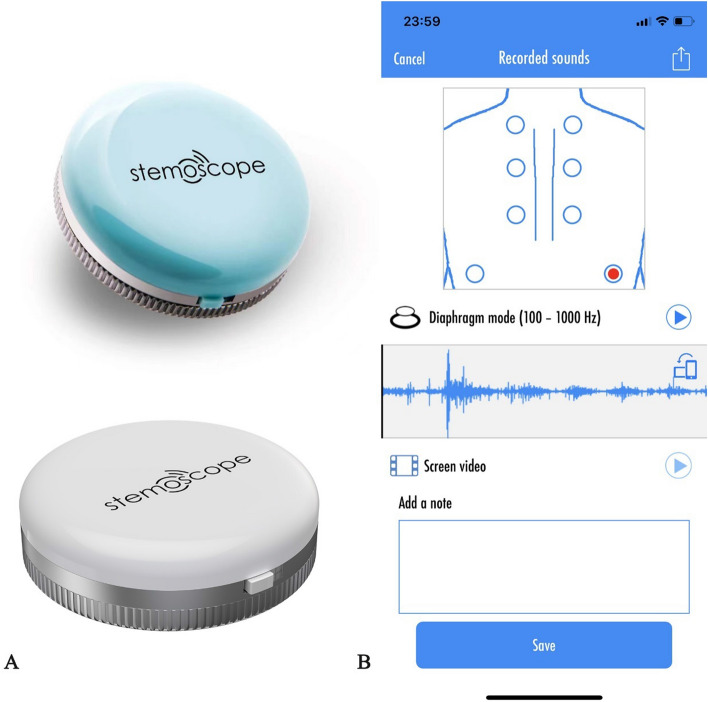
**A** Stemoscope. **B** Stemoscope App to record the respiratory sounds. Tap the red dot on the screen to start recording the sounds. Then the investigators can play the recorded sounds. The App provides powerful options to share recorded sounds, such as Twitter, Facebook, and WeChat

The stethoscope system consists of Bluetooth hardware and App software as previously described [6, 9] (Fig. 4). Stemoscope transmits sound to a smartphone via Bluetooth. The phone processes and amplifies the sound and sends it to a Bluetooth headset in real time. The acquired sound data can be saved. Investigators can then play back the recorded sounds and share them. Four validated stethoscopes were used in this study. Initial device check was performed to ensure the proper work of the stethoscopes. The following criteria were used to determine a device is normal: the battery is full and chargeable; is able to connect to the cellphone; is able to connect to the Bluetooth headset; the heart and lung sounds are clearly audible; and the sounds are able to be stored and played back. All the wireless stethoscopes involved in this study passed the initial check and worked well.

**Fig. 5:**
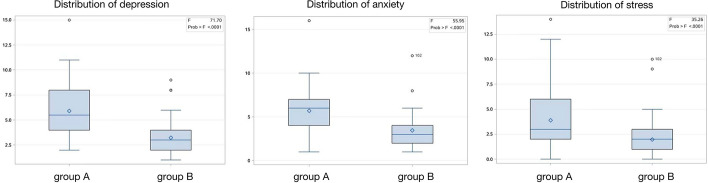
Doctors used Stemoscope to auscultate the patients with COVID-19 in a makeshift hospital. Doctors should wear the Bluetooth headset in advance before putting on the PPE (personal protective equipment)

To capture the acoustic details of auscultation, an extended frequency range mode of 20 to 1000 Hz was used. The upright position was adopted for most patients, with very few patients in an upright or supine position. Prior to the procedure, the patient’s overalls were removed and elevated and the stethoscope was placed against the skin (Fig. 5). To auscultate breath sounds, subjects were asked to take deep breaths (approximately 5 breaths; one breath includes inhaling for 2 s and exhaling for 2 s) and the breath sounds in each part of the lungs were recorded. During the auscultation of cardiac sounds, subjects were asked to breathe calmly. Doctors then made diagnoses simultaneously. At the same time, video and audio files obtained by each patient were also saved, shared, and double checked by two other specialists remotely. Two senior attending doctors Ying Zhuge and Liu Rong (one is a cardiologist and the other is an emergency physician) both worked over 20 years and had experienced auscultatory skills to perform the simultaneous bedside auscultation, and two other specialists (one is a cardiologist and the other is a respiratory specialist) double checked the results remotely. Before the study was began, Ying Zhuge and Liu Rong were trained to manipulate the wireless stethoscopes for 2 weeks and could use them smoothly during the study. The followings were the contents of auscultation: heart and respiratory rate and rhythms, heart normal sounds (S1, S2), heart murmurs and additional sounds, and breath normal sounds and abnormal sounds such as crackles and wheezes. According to the experiences, heart valvular diseases, heart arrhythmia, acute heart failure, and asthmas could be diagnosed by auscultation promptly and accurately as showed in Table 2.

Symptomatic or positive signs and medical history in both groups were recorded, including symptoms, duration, medical interventions, and outcomes. In addition, all subjects were assessed with the Depression, anxiety, Stress Scale (DASS-21) after the consultation.

### Statistical analysis

Categorical variables were presented as frequency rates and percentages and were compared by chi-square or Fisher exact test. Continuous variables are presented as Means ± SD. Means for continuous variables were compared using an independent group t-test or ANOVA when the data were normally distributed; otherwise, the Mann–Whitney test was used. Two-sided *p* < 0.05 was considered statistically significant. All statistical analyses were performed with SPSS software (version 24.0, IBM).

## Data Availability

All relevant data are contained within the article.
