# Correction: The value of the wireless stethoscope in patients with COVID-19 infection in a makeshift hospital

**DOI:** 10.1186/s12938-023-01141-8

**Published:** 2023-08-18

**Authors:** Ying Zhuge, Liu Rong, Lei Ye, Jiaqi Liu, Lingyun Su, Zhiping Zhang, Junshan Wang, Zhi Zhang

**Affiliations:** 1 Department of Cardiology, Shanghai General Hospital, Shanghai Jiaotong University School of Medicine (Originally Named “Shanghai First People’s Hospital”), 100 Haining Road, Shanghai, 200080 People’s Republic of China; 2 Department of Critical Care Medicine, Shanghai General Hospital, Shanghai Jiaotong University School of Medicine (Originally Named “Shanghai First People’s Hospital”), Shanghai, China; 3 Department of Nursing, Shanghai General Hospital, Shanghai Jiaotong University School of Medicine (Originally Named “Shanghai First People’s Hospital”), Shanghai, China; 4 Department of Gastroenterology, Shanghai Tenth People’s Hospital, Tongji University, Shanghai, China

**Correction: BioMedical Engineering OnLine (2023) 22:71** 10.1186/s12938-023-01136-5

Following publication of the original article [1], in this article the figures were interchanged and now the figures are updated correctly.

The original article has been corrected.
